# Mechanochemically Synthesized Nanocrystalline Cu_2_ZnSnSe_4_ as a Multifunctional Material for Energy Conversion and Storage Applications

**DOI:** 10.3390/nano15241866

**Published:** 2025-12-12

**Authors:** Angel Agnes Johnrose, Devika Rajan Sajitha, Vengatesh Panneerselvam, Anandhi Sivaramalingam, Kamalan Kirubaharan Amirtharaj Mosas, Beauno Stephen, Shyju Thankaraj Salammal

**Affiliations:** 1 Research Scholar, Reg.No.20213282132013, Department of Physics and Research Centre, Women’s Christian College, Nagercoil, Affiliated to Manonmaniam Sundaranar University, Abishekapatti, Tirunelveli 629001, India; angelagnes1987@gmail.com; 2 Centre of Excellence for Energy Research, Sathyabama Institute of Science and Technology, Chennai 600119, India; devikasrajan20@gmail.com (D.R.S.); vengatesh.irc@sathyabama.ac.in (V.P.); 3 Department of Physics, Sathyabama Institute of Science and Technology, Chennai 600119, India; anandhis.physics@sathyabama.ac.in; 4 Centre for Functional and Surface-Functionalized Glass, Alexander Dubcek University of Trencín, 911 50 Trencín, Slovakia; kamalan.mosas@tnuni.sk; 5 Department of Physics and Research Centre, Women’s Christian College, Nagercoil, Affiliated to Manonmaniam Sundaranar University, Abishekapatti, Tirunelveli 629001, India

**Keywords:** quaternary chalcogenide, mechanosynthesis, nanocrystalline, semiconductor, kesterite, photo absorbers, energy conversion and storage

## Abstract

Cu_2_ZnSnSe_4_ is a promising light-absorbing material for cost-effective and eco-friendly thin-film solar cells; however, its synthesis often leads to secondary phases that limit device efficiency. To overcome these challenges, we devised a straightforward and efficient method to obtain single-phase Cu_2_ZnSnSe_4_ nanocrystalline powders directly from the elements Cu, Zn, Sn, and Se via mechanochemical synthesis followed by vacuum annealing at 450 °C. Phase evolution monitored by X-ray diffraction (XRD) and Raman spectroscopy at two-hour milling intervals confirmed the formation of phase-pure kesterite Cu_2_ZnSnSe_4_ and enabled tracking of transient secondary phases. Raman spectra revealed the characteristic A_1_ vibrational modes of the kesterite structure, while XRD peaks and Rietveld refinement (χ^2^ ~ 1) validated single-phase formation with crystallite sizes of 10–15 nm and dislocation densities of 3.00–3.20 10^15^ lines/m^2^. Optical analysis showed a direct bandgap of ~1.1 eV, and estimated linear and nonlinear optical constants validate its potential for photovoltaic applications. Scanning electron microscopy (SEM) analysis showed uniformly distributed particles 50–60 nm, and energy dispersive X-ray (EDS) analysis confirmed a near-stoichiometric Cu:Zn:Sn:Se ratio of 2:1:1:4. X-ray photoelectron spectroscopy (XPS) identified the expected oxidation states (Cu^+^, Zn^2+^, Sn^4+^, and Se^2−^). Electrical characterization revealed p-type conductivity with a mobility (μ) of 2.09 cm^2^/Vs, sheet resistance (ρ) of 4.87 Ω cm, and carrier concentrations of 1.23 × 10^19^ cm^−3^. Galvanostatic charge–discharge testing (GCD) demonstrated an energy density of 2.872 Wh/kg^−1^ and a power density of 1083 W kg^−1^, highlighting the material’s additional potential for energy storage applications.

## 1. Introduction

The energy from the sun is one of the most promising sustainable energy sources, with the potential to address a wide range of global challenges. Recent research has concentrated on substituting silicon solar cells with light-absorbing materials due to their superior absorption coefficient, direct bandgap, and relatively low energy and commercial costs. This class of materials may be utilized to develop innovative solar cells. Thin-film technologies utilizing CdTe and Cu (In, Ga) Se_2_ (CIGS) have emerged in recent decades. The efficiency of light-to-electricity conversion for these devices is currently restricted to 25% [1]. Thin-film solar cells are made by depositing thin layers of semiconductor material onto conductive substrates equipped with appropriate electrical connections. The transition from binary to quaternary compounds enables greater flexibility in tailoring material properties and achieving improved device performance [2,3].

Cu_2_ZnSnX_4_ (X = S, Se, and Te) is a quaternary chalcogenide semiconductor that has attracted attention in the field of solar cells. CZTS has the advantage of being composed of completely accessible and non-toxic chemical components, which makes large-scale manufacture easier than with conventional thin-film solar cell absorbers [4,5]. Furthermore, solar cells based on CZTSSe have attained a photoconversion efficiency of up to 14.9%. In 2024, Yimeng Li et al. reported that element inhomogeneity held significant potential in CZTSSe solar cells, with a maximum photoconversion efficiency of 14.9% for CZTSSe [6]. The crystal structure, morphology, stoichiometry, point defect behavior, and their correlations with photovoltaic performance of energy materials remain highly challenging. The current work describes a distinct approach that uses solid-state synthesis for the one-step synthesis of nanocrystalline Cu_2_ZnSnSe_4_ materials [7]; this method does not require solvents and yields high purity and high quantity. Quaternary chalcogenides can be produced by many methodologies. including chemical [8,9,10] and physical methods [11]. Numerous research groups have employed various techniques to develop nanocrystalline semiconductors to reduce fabrication costs and increase the photoconversion efficiency of solar cells and energy storage applications [12,13,14,15]. Recently, researchers have been using quaternary chalcogenides for photoelectrochemical energy conversion [16], supercapacitors [17,18], thermoelectric [19,20], and battery applications [21,22,23]. Quaternary chalcogenide semiconductors are developed through solution processes such as chemical bath deposition [24,25], sol–gel process [26,27], photochemical deposition [28], spray pyrolysis [29,30], chemical vapor depositions [31] and physical vapor deposition techniques like thermal evaporation [32,33], sputtering [34,35], electron beam evaporation [36], and pulsed laser deposition (PLD) [37], atomic layer deposition (ALD) [38] among many others [5,39,40,41].

Quaternary chalcogenides such as CZTS are being studied by several researchers using both vacuum and non-vacuum methods [42]. They essentially reproduce what has been accomplished with CIGS, albeit there are minor variances in the optimal manufacturing conditions. In the last decade, the CIGS market has gained prominence due to its potential for reduced capital expenditures and adaptability in covering large areas. The production and distribution of CZTS and its associated alloys have been complicated by the instability of some metals (Zn and SnS), which may evaporate under reaction conditions. Although element volatility decreases with the production of CZTS, at temperatures above 500 °C, CZTS can convert into binary and ternary compounds and later recrystallize as a quaternary chalcogenide. Several conventional vacuum approaches have been successful. The most efficient CZTS devices were created using particular chemical processes that allow CZTS to grow at low temperatures without causing volatility challenges as well [28,43,44,45]. In 2018, Peter Baláz et al. [46] reported the solvent-free mechanosynthesis of stannite CFTS and kesterite CZTS via the industrial milling process. The process of solid-state mechanochemical reactions resulting in the synthesis of potential absorbers for chalcogenide solar cells. These quaternary sulfide phase evolution, surface composition, solid-state kinetics, and surface morphology were clarified.

Simultaneously, the mixed-valence metal centers and redox-active chalcogen framework make Cu_2_ZnSnSe_4_ a promising electrode material for electrochemical energy storage systems, where stable charge–discharge behavior and structural robustness are required. Despite these dual advantages, achieving phase-pure Cu_2_ZnSnSe_4_ with controlled nanostructure remains challenging, limiting its performance in both photovoltaic and storage applications. In this manuscript, we discuss the solid-state synthesis of stoichiometric Cu_2_ZnSnSe_4_ and its applications in energy conversion and storage. Recently, researchers have focused on more concentrated selenium-based compounds, which provide superior efficiency for mixed ratios. Only a few reports are available in the literature development of Cu_2_ZnSnSe_4_. This study presents a systematic investigation of the mechanosynthesis of Cu_2_ZnSnSe_4_ at varying milling durations_,_ with the resulting materials characterized to elucidate their structural, morphological, optoelectronic, and electrochemical properties. Mechanosynthesis offers a fast, solvent-free, and scalable route for producing phase-pure materials. Cu_2_ZnSnSe_4_ provides a narrow bandgap and higher carrier mobility than CZTS, enabling improved performance.

## 2. Experimental Procedure

**Figure 1:**
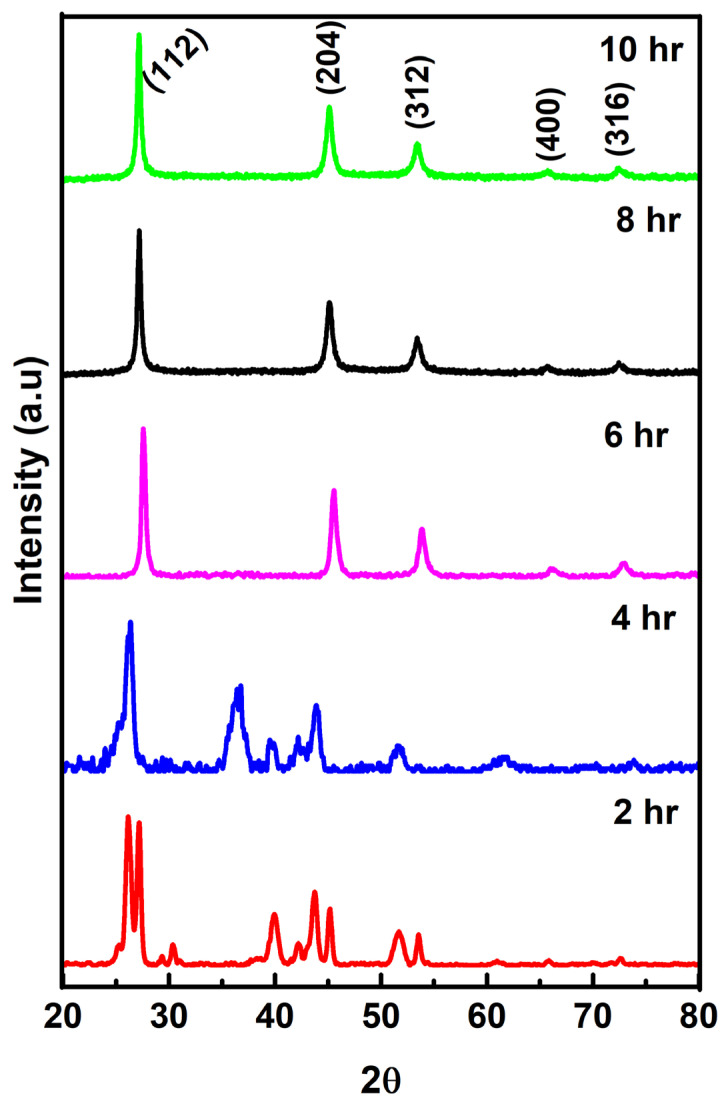
XRD of nanocrystalline Cu_2_ZnSnSe_4_ obtained at various milling times.

The Cu_2_ZnSnSe_4_ nanoparticles were synthesized using SPEX Sample Prep 8000D Mixer (Metuchen, NJ, USA), high-energy ball mill with hardened stainless-steel vials and balls. Several parameters influence the compound formation, such as milling duration, milling type, particle size, milling ball size, and grinding ball type. In our previous investigation, we optimized these parameters, so we ran the experiment for ten hours for the present work. Elemental copper, zinc, tin, and selenium powders (50–100 mesh) were loaded into a stainless-steel vial in a stoichiometric ratio 2:1:1:4. A ball-to-powder weight ratio of 5:1 was used, and milling was carried out at 2750 RPM. Milling was performed continuously for 10 h to promote complete reaction and ensure consistent formation of single-phase Cu_2_ZnSnSe_4_ [7,47]. Small aliquots were extracted every 2 h for XRD analysis to monitor phase evolution shown in Figure 1. After phase confirmation, the 10 h synthesized (0.5 g) powder was compressed into square pellets and annealed at 450 °C for 3 h under 10^−5^ bar vacuum to improve crystalline quality.

The XRD was performed on a BRUKER D8 (Billerica, MA, USA) Advance diffractometer equipped with CuKα radiation (1.54 Å). The diffraction patterns were recorded over 2θ range of 20–80° with a step size of 0.02° and a scan speed of 2° min^−1^. Raman spectroscopy was carried out on a confocal Raman microscope (RENISHAW, New Mills, UK) using a 532 nm excitation laser (50 mW). Spectra were acquired with an acquisition time of 10 s, using a spectral resolution of 0.75 cm^−1^. The laser spot size on the sample surface was approximately 1 μm. The surface morphology and microstructural features of the synthesized materials were examined by FESEM with EDS and HRTEM with SAED (JEOL-2100+, Tokyo, Japan). Optical properties were analyzed using a UV–Vis–NIR spectrophotometer (JASCO, Tokyo, Japan) equipped with a 60 mm integrating sphere. TGA-DSC (NETZSCH STA 449 F3 Jupiter, Selb, Germany) was employed to study the thermal stability of the materials up to 1000 °C. X-ray photoelectron spectroscopy (PHI-VERSAPROBE III, Chanhassen, MN, USA) was used to determine the valence states of materials. The bulk electrical transport properties of Cu_2_ZnSnSe_4_ were measured using a Hall measurement system (ECOPIA HMS-7000: software version 7000, Anyang-si, Republic of Korea). Electrochemical performance was evaluated with a Biologic SP-300 (Thane, India), potentiostat/galvanostatic workstation, equipped with integrated electrochemical impedance spectroscopy (EIS) covering frequency up to 7 MHz and a current range from 500 mA to 10 A.

## 3. Results and Discussions

### 3.1. Structural Analysis

The structural properties of nanocrystalline Cu_2_ZnSnSe_4_ were analyzed using X-ray diffraction tools. The X-pert High Score plus 4.1 software from PANalytical was utilized to analyze the XRD data, which were subsequently compared with the powder diffraction database. The standard 2θ values of the kesterite structure, belonging to the tetragonal crystal system of Cu_2_ZnSnSe_4_, exhibit excellent agreement with JCPDS diffraction data card number 52-0868, as illustrated in Figure 1. Extensive ball milling induces significant alterations in the formation, composition, and crystallinity of the compounds, as demonstrated by XRD evaluation. After up to four hours of milling, diffraction pattern shows secondary phases; Cu_2_ZnSnSe_4_ exhibits peak at 2θ values of 27.2°, 45.1°, 53.4°, 65.7°, and 72.5°, which are associated with the (112), (204), (312), (400), and (316) diffractions of tetragonal system (a = 5.68 Å and c = 11.33 Å).

(1) $$D=\frac{K\lambda }{\left(\beta COS\theta \right)}$$

Scherrer Equation (1) was employed for determining the crystalline size of the material. *D* represents the crystallite size, *K* represents a constant (0.94), *λ* indicates the wavelength (1.5406 Å), *β* indicates the full width at half maximum (FWHM) of the peak, and θ refers to the Bragg angle of the (112) peak. It has been discovered that the Cu_2_ZnSnSe_4_ powders crystallized in the range of ~10–20 nm, and the strains were ~0.04 nm, respectively. The structural formula provided by Williamson and Smallman was utilized to compute the dislocation density of the material (δ = 1/*D*^2^) [48]. The relation (*ε* = *β*_s_/4 tanθ) provided the lattice strain (*ε*), and detailed data were presented in Table 1. The physical characteristics and crystalline size of the absorber layer were shown to be essential for the efficiency of kesterite solar cells [49].

**Figure 2:**
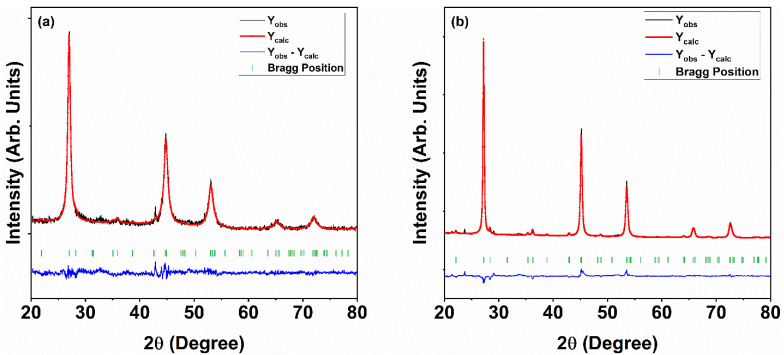
Rietveld refinement of Cu_2_ZnSnSe_4_ (**a**) as-synthesized and (**b**) annealed.

Rietveld refinement is a crucial method in crystallography that facilitates the extraction of intricate structural information from powder diffraction data. This approach has greatly progressed since its debut, enabling improved precision in determining crystal structures and compositions across diverse materials [50]. The crystal structure of Cu_2_ZnSnSe_4_ was analyzed using Rietveld refinement using the FullProf Suite, providing important insights into its structural features and bonding properties (Figure 2). This analysis provided key insights that helped us better understand its potential applications in technology. Throughout the refinement process, we carefully adjusted several structural parameters, including atomic positions, scale factors, lattice parameters, occupancy, and shape functions. The method was used until we reached a sufficient goodness-of-fit value, ideally near 1, or established a high correlation between the observed and calculated patterns. The pseudo-Voigt profile shape function was used consistently throughout the operation. In line with our previous report, all parameters were optimized and the quality of fit to the experimental data was evaluated using multiple metrics, including goodness of fit and several R-factors: R_p_ (profile factor), R_wp_ (weighted profile factor), R_exp_ (expected weighted profile factor), χ^2^ (reduced chi-square), RB (Bragg factor), and RF (crystallographic factor). Table 2 presents the corresponding refinement data.

**Table 1:** Structural characteristics of nanocrystalline compound Cu_2_ZnSnSe_4_.

Sample in h	2θ (Degree)		d-Spacing (Å)		(hkl)	Crystallite Size (nm)	Dislocation Density (δ) × 10^15^ Lines/m^2^	Strain	Lattice Parameters (a = b ≠ c) (Å)			
									Exp		Cal	
	Exp	Cal	Exp	Cal					a	c	a	c
6	27.37	27.1	3.236	3.283	(112)	18.6	2.8	0.031	5.67	11.26	5.77	11.48
8	27.2	27.1	3.245	3.283	(112)	19.6	2.58	0.030	5.68	11.28	5.77	11.48
10	27.16	27.1	3.278	3.283	(112)	19.9	2.57	0.030	5.68	11.29	5.77	11.48

**Table 2:** Refinement data of Cu_2_ZnSnSe_4_ as-synthesized and annealed.

Empirical Formula	Cu_2_ZnSnSe_4_ (As Synthesized)	Cu_2_ZnSnSe_4_ (Annealed)
Formula weight (g/mol)	627.03	627.03
Temperature	RT	RT
Wavelength	*λkα*1 = 1.54056 *λ kα*2 = 1.54439	*λkα*1 = 1.54056 *λ kα*2 = 1.54439
2θ step scan increment	0.020006	0.020007
2θ range (°)	Min = 20.120001	Min = 20.120001
	Max = 90.000000	Max = 90.000000
Program	Fullprof	Fullprof
Zero-point (2θ)	0.1732	0.1503
Pesudo-Voigt function PV = ηL + (1 − η) G	η = 0.1923	η = 0.0906
Caglioti parameters	U = 0.08682	U = 0.97014
	V =−0.11404	V = −0.43220
	W = 0.09554	W = 0.29198
No. of refined parameters	11	14
Crystal system	Tetragonal	Tetragonal
Space group	I$\bar{4}$	I$\bar{4}$
a (Å)	5.6552	5.6654
b (Å)	5.6552	5.6654
c (Å)	11.2679	11.4533
V (Å) 3	360.365	367.739
Z	4	4
Atom number	16	16
R_p_	4.85	8.15
R_wp_	6.45	10.5
R_exp_	5.06	8.51
χ^2^	1.62	1.52

### 3.2. Raman Spectroscopy

**Figure 3:**
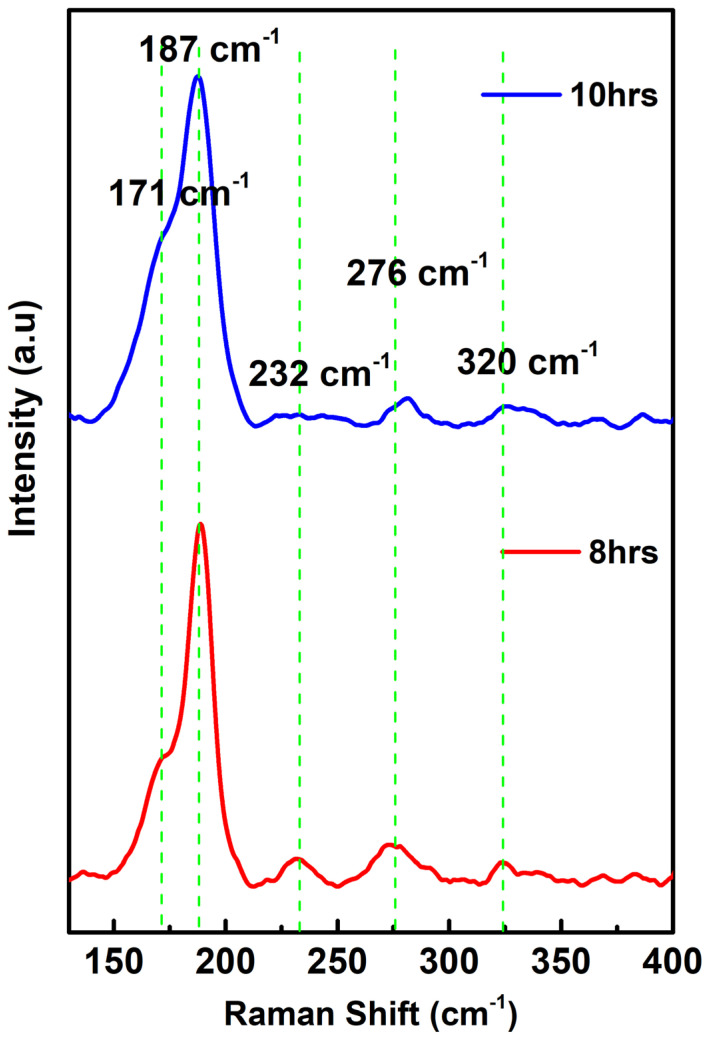
Raman vibrational modes of Cu_2_ZnSnSe_4_ nanocrystals.

Raman spectroscopy of the Cu_2_ZnSnSe_4_ (quaternary chalcogenide) compound was performed using a 532 nm excitation laser focused onto the sample surface. To ensure accurate peak positioning, all spectra were calibrated using the first-order Raman spectrum of monocrystalline silicon at 520 cm^−1^. The Cu_2_ZnSnSe_4_ samples exhibited distinct Raman vibrational bands at 187, 232, 276, and 320 cm^−1^. The Raman spectra show a peak at 187 cm^−1^, indicating the most prevalent A1 vibrational modes of kesterite-structured Cu_2_ZnSnSe_4_, arising primarily from Se anion vibrations against the nearly stationary cation sublattice. The sharp and intense nature of this peak confirms successful crystallization of the mechanosynthesized Cu_2_ZnSnSe_4_ and indicates the formation of a single-phase quaternary compound semiconductor. The significant peak at 192 cm^−1^ corresponds to Cu_2_ZnSnSe_4_ A1 vibrational mode, which is presumably caused by selenide vibrations shown in Figure 3. The vibrations of quaternary chalcogenides range from 170 to 200 cm^−1^ and are attributed to local cation disorder and defect-induced perturbations within the lattice.

A weaker Raman mode at ~232 cm^−1^ is assigned to the E/B symmetric vibrational modes of the kesterite structure. Additionally, a vibrational mode around ~216 cm^−1^, associated with Cu, Zn, and Sn cations against Se, aligns well with previously reported Raman modes of Cu_2_ZnSnSe_4_. Higher wave number peaks at ~276 and ~320 cm^−1^ further corroborate the presence of kesterite-type lattice dynamics [51,52].

### 3.3. Optical Properties

**Figure 4:**
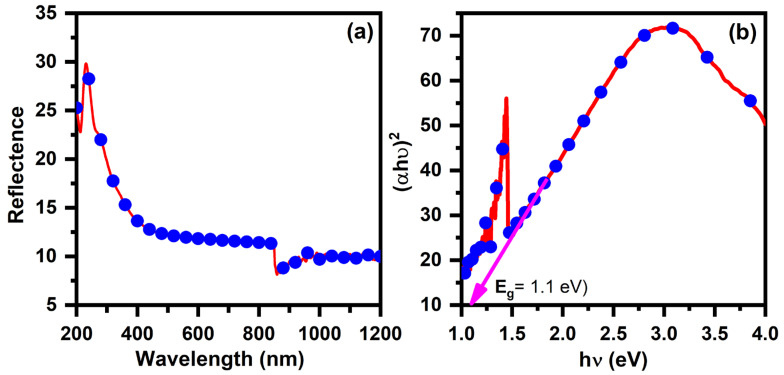
(**a**,**b**) Optical properties of Cu_2_ZnSnSe_4_ nanocrystals by mechanosynthesis.

The optical absorption spectra in the visible and near-infrared areas are associated with electronic transitions, essential for understanding the electronic band structure of semiconducting materials. A UV–Vis spectrophotometer measured the optical absorbance spectra of Cu_2_ZnSnSe_4_ materials at ambient temperature across the 300–1100 nm wavelength range. Optical investigations reveal direct band-to-band transitions in these semiconductors. The standard relationship for semiconductor near-edge optical absorption was utilized to examine the absorption edge (Figure 4a). A Tauc plot is created, a graph of hv against (αhv)^2^, to examine the direct bandgap. The extrapolation of the linear component to the energy axis yields an intercept on the X-axis, representing the optical bandgap. Figure 4b depicts the optical band gap of Cu_2_ZnSnSe_4_ at ambient temperature, recorded at 1.1 eV. The E_g_ values for Cu_2_ZnSnSe_4_ in this investigation are slightly lower than the bulk value. This corresponds with prior findings and may be ascribed to the material nanocrystalline nature [53,54].

**Figure 5:**
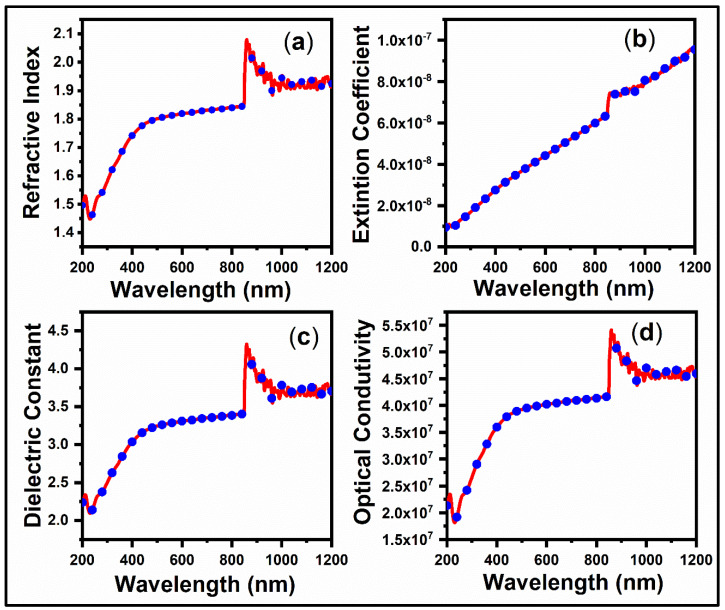
(**a**–**d**) Optical Properties of Cu_2_ZnSnSe_4_ nanocrystals by mechanosynthesis.

The optical constants were evaluated using Equation (2) to investigate the optical and electronic responses of the synthesized semiconductor nanocrystals. The refractive index, which characterizes the phase velocity of light in a medium, was determined accordingly. The observed increase in refractive index can be attributed to enhanced surface scattering arising from increased surface roughness, which effectively reduces the mean free path of charge carriers. The variation in refractive index (*n*) with wavelength (*λ*) is presented in Figure 5a.

(2) $$n=\frac{1+\sqrt{R}}{1-\sqrt{R}}$$

Figure 5b shows the extinction coefficient (*k*) of the mechanosynthesized Cu_2_ZnSnSe_4_ for the wavelength. This indicates that the amount of light absorbed by the substance was computed using the corresponding formula (Equation (3)).

(3) $$k=\frac{\alpha \lambda }{4\pi }$$

The real (*ε*_1_) (Equation (4)) and imaginary (*ε*_2_) (Equation (5)) parts of the optical dielectric constant of mechanosynthesized Cu_2_ZnSnSe_4_ were evaluated using the following Equations (4) and (5), and the results are displayed in Figure 5c. It revealed that the dielectric constant increased with respect to wavelength. The complex number known as the dielectric function represents how a substance reacts to electromagnetic radiation. The phase velocity of light is determined by the material refractive index, which is represented by the real component (*ε*_1_). The material absorption or extinction coefficient, represented by the imaginary component (*ε*_1_), specifies the amount of light absorbed during propagation.

(4) $$\varepsilon_{1}=n^{2}-k^{2}$$

(5) $$\varepsilon_{2}=2nk$$

The optical conductivity (*σ*_0_) of Cu_2_ZnSnSe_4_ was calculated using Equation (6). Where *α* denotes the absorption coefficient of the mechanosynthesized semiconductor, *n* is the refractive index, and c is the speed of light. The dependence of *σ*_0_ on the wavelength is depicted in Figure 5d. The results confirm that Cu_2_ZnSnSe_4_ exhibits photoinduced conductivity, indicating its ability to absorb incident photons and convert them into electronic excitations rather than transmitting the light.

(6) $$\sigma_{0}=\frac{\alpha nc}{4\pi }$$

The results indicate that the optical characteristics of the analyzed materials, such as bandgap, absorption coefficient, and optical responses, imply their enhanced suitability for use as light absorbers. Researchers often examine optical conductivity to improve their knowledge of many material properties, including electronic structure, energy band gaps, and carrier transport behavior.

### 3.4. X-Ray Photoelectron Spectroscopy Analysis of Cu_2_ZnSnSe_4_

**Figure 6:**
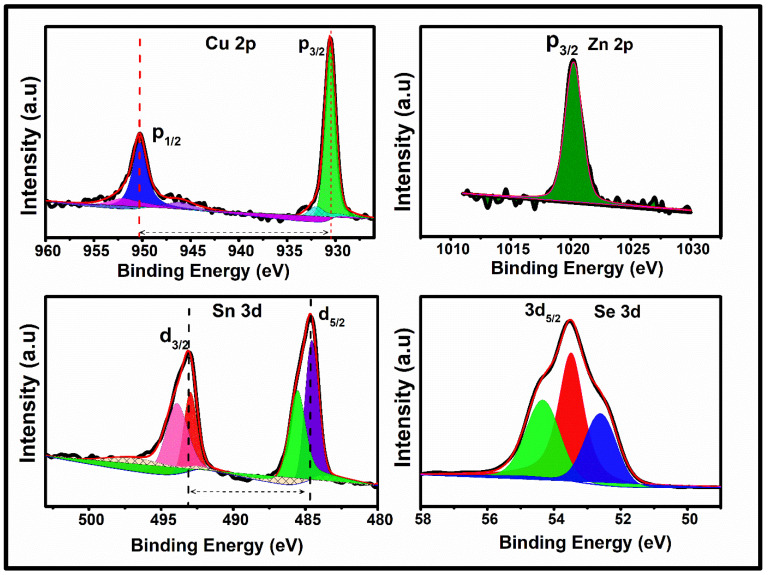
XPS analysis of Cu_2_ZnSnSe_4_ nanocrystals by mechanosynthesis.

The valence states of all four constituent elements in the Cu_2_ZnSnSe_4_ powder were examined using X-ray photoelectron spectroscopy (XPS), and deconvolution was performed using the CASA XPS software, as shown in Figure 6 [55]. The Cu+ oxidation state is confirmed by the Cu 2p_3/2_ peaks at 931.6 eV and Cu 2p_1/2_ at 951.7 eV, respectively, with a spin–orbit splitting value of 20.1 eV, characteristic of Cu (I) species. The Zn 2p spectrum exhibits a single Zn 2p_3/2_ peak at 1020.4 eV, consistent with the Zn^2+^ valence state. The Sn 3d_3/2_ and 3d_5/2_ peaks at 493.0 eV and 484.5 eV, respectively, along with a spin–orbit separation of 8.5 eV, confirm the presence of Sn^4+^ rather than Sn^2+^. A subtle shoulder feature adjacent to the main Sn 3d peak further indicates slight surface-related variations typical of Sn^4+^ environments in chalcogenides. The Se^2−^ spectrum displays the main Se 3d_5/2_ peak at 53.5 eV, indicative of Se^2−^, accompanied by a minor shoulder attributed to multiplet interactions commonly observed in selenide systems. Overall, the XPS results confirm the expected oxidation states of Cu^+^, Zn^2+^, Sn^4−^, and Se^2−^, validating the successful formation of the quaternary Cu_2_ZnSnSe_4_ phase.

### 3.5. Thermal Analysis of Cu_2_ZnSnSe_4_

**Figure 7:**
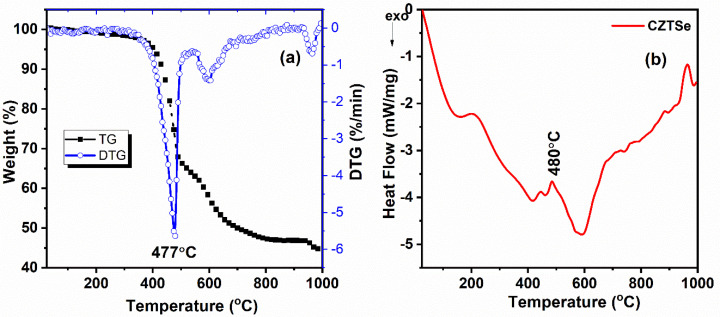
Thermal behavior of Cu_2_ZnSnSe_4_ nanocrystals by mechanosynthesis (**a**) TG/DTG, (**b**) DSC.

Thermo-gravimetry/differential thermal analysis (TG/DTA) quantifies the thermal effects associated with physical or chemical transformations in relation to temperature or time. The material is uniformly heated in a vacuum or in the presence of a gas medium, specifically Argon. TGA was employed to examine the thermal degradation of Cu_2_ZnSnSe_4_ powder up to 1000 °C. The Cu_2_ZnSnSe_4_ materials underwent heating in a high-temperature furnace under an argon environment, increasing from ambient temperature to 1000 °C at a rate of 10 °C per min. The primary weight loss was observed at 477 °C, reaching up to 55%. The minimal residue in the materials is indicated by the TG graph in Figure 7a. The thermal stability of the materials was evaluated using differential scanning calorimetry (DSC), as presented in Figure 7b [55]. The exothermic peak indicates that the materials begin to melt at 480 °C, demonstrating that the synthesized materials do not exhibit a sharp, well-defined melting point.

### 3.6. Surface Analysis of Cu_2_ZnSnSe_4_ Using FESEM

**Figure 8:**
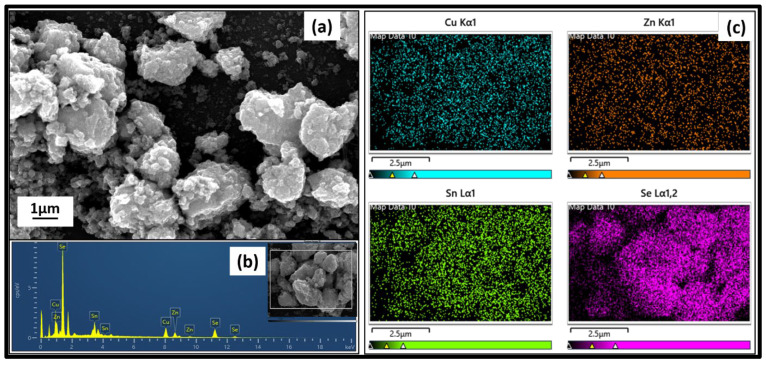
FESEM image of Cu_2_ZnSnSe_4_ nanocrystals by mechanosynthesis: (**a**) surface morphology, (**b**) EDAX, and (**c**) elemental mapping of Cu, Zn, Sn, and Se.

Cu, Zn, and Sn in their metallic forms can be used as precursors for mechanosynthesis to produce Cu_2_ZnSnSe_4_ powder. The surface properties of the synthesized materials were studied using a field emission scanning electron microscope (FESEM). We found that the nanocrystalline material was bonded together to form clusters at the nanometer scale, as illustrated in Figure 8a. FESEM images demonstrate low and high magnifications of samples milled for 10 h. Figure 8a indicates that the sample comprises particles of various sizes, which tend to form micro agglomerates. Larger magnification enables the tracing of sub-micrometer particles. These agglomerates are made up of nanoparticles, namely, nanocrystals. Higher magnification can also be utilized to observe this pattern in flat nanoparticles. Furthermore, the elemental composition was identified by energy-dispersive X-ray analysis. The materials’ stoichiometric ratio is (2:1:1:4), as shown in Figure 8b. The elemental mapping was conducted to determine the uniform distribution of the elements in the nanocrystalline powder depicted in Figure 8c, and we discovered that the materials crystallized in a single phase with no detectable impurities [15].

### 3.7. Surface Analysis of Cu_2_ZnSnSe_4_ Using HRTEM

**Figure 9:**
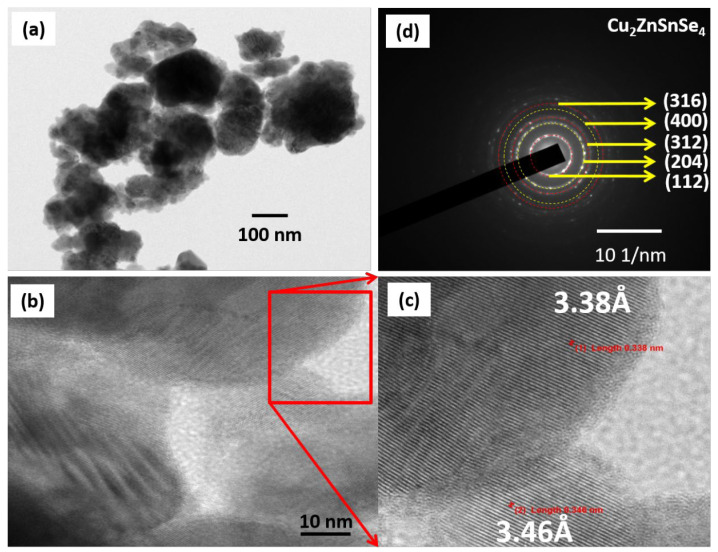
HRTEM image of Cu_2_ZnSnSe_4_ nanocrystals by mechanosynthesis (**a**), surface morphology (**b**,**c**)), high-resolution image, and (**d**) SAED pattern.

HRTEM examination was utilized to study the precise structural information of the Cu_2_ZnSnSe_4_. Figure 9a–c displays the HRTEM pictures of excellent crystallographic faces, corresponding to the (112) planes of Cu_2_ZnSnSe_4_ (Materials Project reference). Moreover, we evaluated interplanar spacing from the SAED using ImageJ software, and the values are tabulated. After using ImageJ software to compare the interplanar spacing, which is ~3.42 Å for the (112) plane from SAED, the findings are reported in Table 3. The SAED pattern confirms that the produced powder exhibits excellent crystalline quality, as shown in Figure 9d. Furthermore, the absence of mixed impure phases confirms the purity of the kesterite Cu_2_ZnSnSe_4_ phase. The HRTEM image helps us to determine the crystalline lattice defects, including vacancies, dislocations, and antisite defects within the crystal lattice.

### 3.8. Electrical Properties of Cu_2_ZnSnSe_4_ by Hall Effect Measurement

The electrical properties of Cu_2_ZnSnSe_4_ were examined at room temperature using the Hall effect in van der Pauw configuration to investigate resistivity, carrier mobility, and carrier concentration. A dense square pellet (0.5 mm thickness) was fabricated by compressing 0.5 g of the mechanosynthesized Cu_2_ZnSnSe_4_ powder under high pressure using a hydraulic press, ensuring enhanced mechanical stability and uniform current distribution across the sample. Hall measurement was conducted by applying a current of 20 mA under a 0.5 Tesla. The materials exhibited clear p-type conductivity, as evidenced by the positive Hall coefficient, confirming that holes are the dominant charge carriers responsible for electrical transport. The extracted electrical parameters include a carrier mobility (μ) of 2.09 cm^2^/Vs, sheet resistance (ρ) of 4.87 Ω cm, and carrier concentration (1.23 × 10^19^ cm^−3^). The solvent-free mechanosynthesis route contributes to improved crystallinity by minimizing grain disorder and enhancing intergranular connectivity, thereby supporting efficient charge transport in Cu_2_ZnSnSe_4_.

### 3.9. Electrochemical Analysis

**Figure 10:**
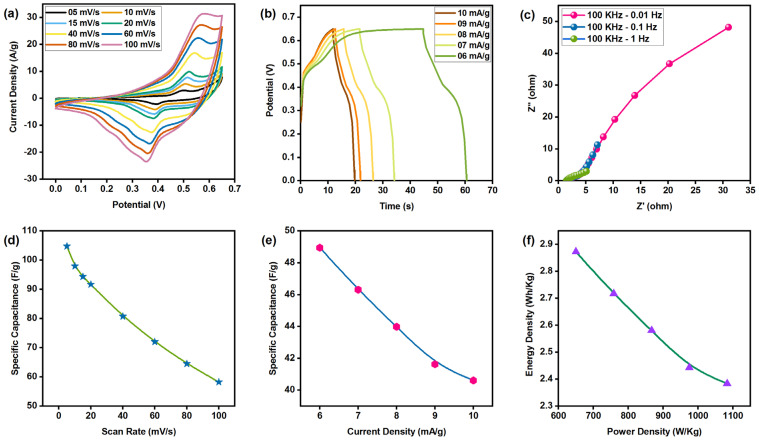
Electrochemical analysis of Cu_2_ZnSnSe_4_ nanostructures: (**a**) CV curve, (**b**) GCD curves, (**c**) EIS spectrum, (**d**) scan rate vs. specific capacitance, (**e**) current density vs. specific capacitance, and (**f**) specific capacitance vs. energy and power density.

The electrochemical performance of the synthesized Cu_2_ZnSnSe_4_ nanocrystals was evaluated in 2 M KOH electrolyte using cyclic voltammetry (CV), galvanostatic charge–discharge (GCD), and electrochemical impedance spectroscopy (EIS). Cyclic voltammetry was employed to probe the redox behavior and electron transfer processes, where the current response is recorded as a function of the applied potential at varying scan rates. The cyclic voltammetry technique measures current as the electric potential varies with the associated scan rates. Figure 10 presents the CV, GCD, and EIS profile of Cu_2_ZnSnSe_4_ electrodes measured over a potential window of 0–0.65 V at scan rates ranging from 5−100 mV/s. Electrodes were fabricated following a procedure adapted from the standard protocol. The active material mixture consisted of 80 wt% Cu_2_ZnSnSe_4_, 10 wt% acetylene black (conductive additive), and 10 wt% polytetrafluoroethylene (PTFE) binder. The next step entailed creating a slurry paste by gradually adding n-methyl pyrrolidone (NMP) by ball milling. The nickel foam current collector (2 × 1 cm) was cut using a standard die cutter and sequentially cleaned in ethanol, deionized water, and diluted HCl to remove surface contaminants, followed by drying in a vacuum oven. The slurry paste was uniformly applied to nickel foam with an active area of 1.0 cm^2^ using a doctor blade method. The coated electrodes were dried at 80 °C for 12 h under vacuum to remove residual solvent and then pressed to enhance adhesion and improve mechanical stability. The final active mass loading of Cu_2_ZnSnSe_4_ on each electrode was 6.3 mg. Electrochemical measurements were conducted using a three-electrode configuration, with Ag/AgCl serving as the reference electrode and a platinum wire as the counter-electrode.

Cyclic voltammetry graphs of semiconductor nanocrystals demonstrate a displacement of redox peaks with elevated scan speeds. This results from irreversible electrode reactions occurring at elevated scan speeds due to an electrode polarization effect. Cyclic voltammetry curves were employed to evaluate specific capacitance at different scan rates. At a scan rate of 5 mV/s, the specific capacitance of quaternary chalcogenide semiconductor nanocrystals is around 104.769 F/g; however, it decreases with an increase in scan rate. Linear charge–discharge curves exhibited excellent capacitive performance, achieving a maximum specific capacitance of 48.953 F/g at a current density of 6 mA/g, according to GCD analysis. The Cu_2_ZnSnSe_4_ electrode achieved a maximum energy density of 2.872 Wh/kg and a power density of 1083 W/kg, as demonstrated by GCD profiles. Attention to electrochemical impedance is essential for comprehending the electrochemical properties of electrode materials over a wide frequency range (0.1 Hz to 1 MHz). This clarifies the reduced interfacial charge transfer resistance at the electrode–electrolyte interface. Enhanced conductivity and diminished interfacial charge transfer resistance are anticipated to augment capacitive performance and charge carrier mobility in solution. The electrochemical characteristics of Cu_2_ZnSnSe_4_ make it appropriate for the production of large-scale energy storage and electronic devices.

## 4. Conclusions

Cu_2_ZnSnSe_4_ has been validated as a promising multifunctional material for energy conversion and storage. Nanocrystalline Cu_2_ZnSnSe_4_ powders were successfully synthesized via a single-step mechanochemical route using elemental Cu, Zn, Sn, and Se in a 2:1:1:4 ratio. The material crystallized directly into the tetragonal kesterite phase, with a crystallite size of 10 to 20 nm. Structural analysis by HRTEM revealed well-defined lattice fringes, while SAED patterns showed concentric rings consistent with XRD results and the literature. Raman spectroscopy confirmed the characteristic longitudinal-optical modes of Cu_2_ZnSnSe_4_, and XPS verified the expected oxidation states (Cu^+^, Zn^2+^, Sn^4+^, and Se^2−^), together demonstrating high-phase purity. The synthesized Cu_2_ZnSnSe_4_ exhibited a direct band gap of 1.1 eV, and optical constants aligned with reported values, confirming its suitability as an efficient photoabsorber. Hall measurement indicated p-type conductivity with carrier concentration and mobility parameters favorable for optoelectronic applications. In addition to photovoltaic relevance, electrochemical studies revealed stable redox behavior and appreciable charge-storage capacity, highlighting its potential in energy storage systems. The favorable band alignment and minimal conduction-band offset highlight opportunities for further performance optimization through controlled synthesis. Overall, the ability to obtain phase-pure nanocrystalline Cu_2_ZnSnSe_4_ via a simple mechanosynthesis approach, coupled with its advantageous optical, structural, and electrochemical properties, reinforces its promise for next-generation thin film photovoltaic and hybrid energy storage technologies.

## Figures and Tables

**Table 3:** Structural parameters of Cu_2_ZnSnSe_4_ nanocrystals by mechanosynthesis observed from the SAED.

Label	1/2r (nm^−1^)	1/r (nm^−1^)	r (nm)	d—Spacing (Å)		(hkl)
				TEM (Obs)	Standard	
1	5.844	2.922	0.342	3.42	3.31	(112)
2	9.742	4.871	0.205	2.05	2.08	(204)
3	11.518	5.759	0.173	1.73	1.736	(312)
4	13.442	6.721	0.148	1.48	1.442	(400)
5	15.195	7.5975	0.131	1.31	1.312	(316)

## Data Availability

The original contributions presented in this study are included in the article. Further inquiries can be directed to the corresponding authors.
